# The mental health impacts of health and human service work: Longitudinal evidence about differential exposure and susceptibility using 16 waves of cohort data

**DOI:** 10.1016/j.pmedr.2019.100826

**Published:** 2019-02-23

**Authors:** Allison Milner, Tania L. King, Anne Kavanagh

**Affiliations:** a Disability and Health Unit, Centre for Health Equity, School of Global and Population Heath, University of Melbourne, Melbourne, Victoria, Australia; b Melbourne Disability Institute, The University of Melbourne, Victoria 3010, Australia

**Keywords:** Carers, Health professionals, Job stressors, Mental health, Longitudinal data analysis

## Abstract

The health and human care workforce comprise a substantial and increasing proportion of the employed population in high income countries. This diverse workforce is comprised of high skilled workers, such as doctors and nurses, as well as lower skilled workers such as carers and support workers. This paper assessed psychosocial working conditions among health and human care workers compared to other workers. We also examined the effects of psychosocial working conditions on mental health. The data source was 16 waves of the Household Income Labour Dynamics in Australia survey. The exposure was a multidimensional, previously validated psychosocial job quality index. The outcome was changes in the Mental Health Inventory-5 (MHI-5). The effect modifier was a multicategory health and human care occupational variable. Random and fixed effects linear regression models were used to unpack between- versus within- person differences. Time varying confounders were controlled for. We found evidence of effect modification. Carers and support workers experienced a 4.90-point decline (95% CI −6.23 to 3.57) on the MHI-5 when reporting 3 or more job stressors compared to no stressors. These workers also reported lower levels of mental health than other occupational groups and had greater exposure to poor psychosocial working environments. Health workers also reported substantial declines on the MHI-5 when exposed to 3 or more job stressors (−3.50, 95% CI −5.05 to −1.94). Understanding the quality of employment in this workforce, and consequent impacts of this employment on mental health is critical to ensuring sustainable individual, organizational and client-related outcomes.

## Highlights

• The health and human service workforce is rapidly increasing in high-income countries.
• The diverse workforce includes both high-skilled and lower skilled workers.
• Lower skilled workers are differentially exposed to poor quality working conditions.
• All workers have a decline in mental health in response to poor quality work.
• This decline is particularly apparent in lower skilled workers.

## 1 Introduction

Across high-income countries, the ageing population and increasing burden of mental health problems has increased the demand for health and human service work (Rechel et al., 2009). This diverse workforce includes health professionals such as doctors and nurses, as well as social, disability and elderly care workers (Madsen et al., 2012; Wieclaw et al., 2006). A common feature of all health and human care work is that it involves a high degree of person-related psychological or physical help or care (Dollard et al., 2000) in the home (e.g., personal care attendants and home care workers) or within health care facilities or hospitals (e.g., nurses, doctors, allied health care workers). Internationally, the characteristics of the workforce indicate it is highly feminized (Hewko et al., 2015).

Health and human care workers are exposed to specific psychosocial working stressors such high emotional demands (Dollard et al., 2000; Madsen et al., 2010; van Vegchel et al., 2004), threats of violence and violence itself (Aagestad et al., 2016), as well as high job demands and low job control (Gray-Stanley and Muramatsu, 2011; Vassos et al., 2019). However, it is likely that there is variation in exposure to stressors across different jobs within health and human care work. For example, a recent review on healthcare aides (paid caregivers for persons with disabilities and older persons) highlighted poor pay, lack of clarity of work roles and job insecurity as risk factors for occupational injury and turnover (Hewko et al., 2015). This may be different from higher-skilled hospital nurses and physicians, where other stressors including low control, high demands and lack of social support have been identified as prominent job stressors (Weigl et al., 2013).

Adverse working conditions associated with health and human care work have been linked to a number of poor outcomes, including burnout (Gray-Stanley and Muramatsu, 2011; Innstrand et al., 2004; Borritz et al., 2005), sickness absence (Aagestad et al., 2016), and turnover (Mazurenko et al., 2015). European register-based studies have documented a relationship between health and human care work and antidepressant use (Madsen et al., 2012; Madsen et al., 2010; Buscariolli et al., 2018) and diagnosed affective disorder (Wieclaw et al., 2006). However, within this, there are substantial differences depending on the specific job undertaken. Buscariolli et al. (2018), noted that social workers had a much greater likelihood of antidepressant use than health professionals such as doctors. Compared to medical doctors, nurses and social workers had a significantly elevated risk of developing an affective disorder (Wieclaw et al., 2006).

This present study seeks to advance knowledge about working conditions and mental health among the health and human service workers using a longitudinal research design. As it stands, longitudinal studies examining the working conditions of health and human care workers are limited and, with the exception of a few studies (Wieclaw et al., 2006; Madsen et al., 2010; Buscariolli et al., 2018), do not make comparison to other occupational groups. Longitudinal studies that do exist have focused on health service engagement (i.e., measured through antidepressant use and hospital presentations for depression) rather than the broader construct of mental health, and thus only cover that segment of the population that seek treatment.

The study has three main aims. First, it will examine psychosocial working conditions among health and human care workers in relation to other workers to understand differential exposure. Second, it will evaluate the effect of psychosocial working conditions on changes in mental health among health and human care workers and make comparison to other workers to understand differential susceptibility. Third, the paper will assess how much of the relationship between psychosocial working conditions and changes in mental health may be due to within person effects, thus providing stronger evidence about the role of psychosocial working conditions on changes in mental health.

## 2 Methods

### 2.1 Data source

The Household, Income and Labour Dynamics in Australia (HILDA) survey is a longitudinal, nationally representative study of Australian households established in 2001. Research staff collect detailed information annually from over 13,000 individuals within over 7000 households (Wilkins, 2013). The response rate to wave 1 was 66% (Wilkins, 2013). The survey covers a range of dimensions including social, demographic, health and economic conditions using a combination of face-to-face interviews with trained interviewers and a self-completion questionnaire.

**Fig. 1:**
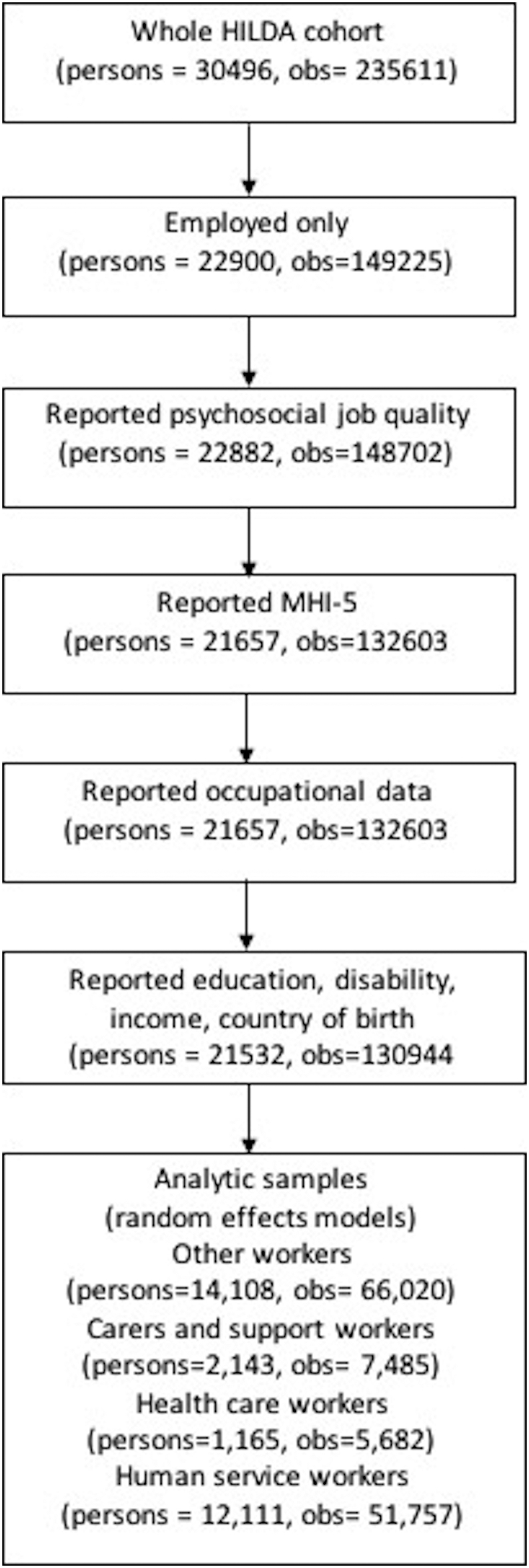
Study sample, HILDA, 2001 to 2016.

The initial wave of the survey began with a large national probability sample of Australian households occupying private dwellings (Wilkins, 2013). Interviews were sought in later waves with all persons in sample households who turned 15 years of age. Additional persons have been added to the sample as a result of changes in household composition. Inclusion of these new households is the main way in which the HILDA survey maintains sample representativeness. A top-up sample of 2000 people was added to the cohort in 2011 to allow better representation of the Australian population using the same methodology as the original sample (i.e., a three-stage area-based design) (Watson, 2011). The response rates for the HILDA survey are above 90% for respondents who have continued in the survey and above 70% for new respondents being invited into the study (Wilkins, 2013). The main variables examined in this study were available in all annual waves of HILDA (2001 to 2016). The flow of people into the analytic sample can be seen in Fig. 1.

### 2.2 Outcome variable

Mental health was assessed using the five-item Mental Health Inventory (MHI-5), a subscale from the Short Form-36 (SF-36) general health measure. The MHI-5 assesses symptoms of depression and anxiety (nervousness, depressed affect) and positive aspects of mental health (feeling calm, happy) in the past 4 weeks. The MHI-5 has reasonable validity and is an effective screening instrument for mood disorders or severe depressive symptomatology in the general population (Rumpf et al., 2001; Yamazaki et al., 2005; Gill et al., 2006) and has been validated as a measure for depression using clinical interviews as the gold standard (Rumpf et al., 2001; Berwick et al., 1991; Cuijpers et al., 2009). The current analyses use the continuous MHI-5 score (scale 1 to 100), with higher scores representing better mental health. Although there is no universally accepted translation of MHI-5 score difference to clinical meaningfulness, a difference of four or more points on the norm based scale (T-score) has been suggested to reflect a minimally important difference (McHorney et al., 1993).

### 2.3 Effect modifier

We looked at a four-level categorical variable representing whether a person was employed in health and human care occupation, based on the Australian and New Zealand Standard Classification of Occupations (ANZSCO) at the two digit level (ABS, 2013). These categories were carers and support workers (e.g., health and welfare support workers, carers and aides) and health care workers (e.g., allied health professionals, health diagnostic and promotion, health therapy, medical practitioners, nursing and midwifery). We made comparison to human service workers, comprised of other occupations that have a high-degree of non-health related person contact such as clerical and administrative workers (e.g., clerical and administration, personal assistants, general clerical, inquiry clerks, numerical clerks), sales workers, hospitality workers (e.g., legal, social and welfare professionals), those employed in protective services (e.g., protective service officers). Our reference category was non-human service workers (e.g., all other occupations than those in other categories). The overall description of the specific occupations included in each of these categories can be seen in Supplementary Table 1.

### 2.4 Exposure variable

The exposure was a multidimensional measure of psychosocial job quality assessing four main perceived job stressors: control, demands and complexity, job insecurity, and unfair pay (Butterworth et al., 2011a; Butterworth et al., 2011b; Leach et al., 2010). Full details of the construction and validation of the job quality measure are presented elsewhere (Butterworth et al., 2011a; Butterworth et al., 2011b; Leach et al., 2010). In brief, factor analysis and structural equation modelling identified three separate factors, which were labelled: job demands and complexity (three items); job control (three items); and perceived job security (three items). An additional single item assessing whether respondents considered that they were paid fairly for their efforts at work was included as a fourth factor measuring an important aspect of the effort-reward imbalance model (Siegrist et al., 2004). The individual scales are similar to widely used measures of job demands and control, and other employment conditions such as casual status, hours worked and shift work (Butterworth et al., 2011a; Butterworth et al., 2011b; Leach et al., 2010). Each factor was dichotomized at the quartile to identify those experiencing the greatest adversity and the composite measure constructed by summing the number of adverse psychosocial job conditions (high job demands and complexity, low job control, high job insecurity and unfair pay). Because of the small number of respondents reporting all four job adversities in a single year/wave, this composite scale was top-coded at three and, thus, produced four categories ranging from optimal jobs to three or more psychosocial adversities (poorest quality jobs). In this study, we used the overall index, scored from no psychosocial job stressors (0) to three or more stressors.

### 2.5 Other variables

Our confounders included age (18 to 64 years, measured continuously), education (less than high school, high school, diploma or certificate, bachelors degree and postgraduate), household structure (couple without children, couple with children, lone parent with children, lone person, and multiple other people), and weekly household income (equivalised). Household income calculated by summing the income components for all adults in the household, with imputed values computed for missing variables using nearest neighbour imputation (20% imputed values for observations in the sample) (Watson, 2004; Little and Su, 1989). Household disposable income was then equivalised using the modified OECD scale (Haagenars et al., 1994) and converted to national quintiles using statistics published annually by the Australian Bureau of Statistics. Other confounders considered was employment arrangement (permanent, casual/labour hire, fixed term, and self-employed), long-term health conditions (disability) (yes or no), country of birth (Australia, English speaking, non-English speaking), and gender (male or female).

### 2.6 Analysis

First, our analysis assessed: 1) differences in psychosocial job stressors between health and human care work and other workers, and; 2) heterogeneity in the associations between psychosocial job stressors and mental health between those working as health and human care workers and other occupational groups. We first used descriptive analysis (frequency and means) across the whole analytic sample to examine these aims. Following this, we used random effects longitudinal regression models to assess differences in the relationship between psychosocial job stressors and mental health between occupational groups. We ran a model with an interaction term in the model (health and human care occupation*psychosocial job stressor). The results of this model were compared to a main effects model using the likelihood ratio test. The occupational variable was time varying in this analysis. Once significant differences were established, we stratified the models by occupational group (i.e., the effect modifier), which was thus kept constant or time-invariant. In all models, we controlled for relevant confounders. Estimated marginal means and 95% confidence intervals were calculated for the effects of psychosocial job quality on the MHI-5. We conducted a sensitivity analysis looking at each of the individual job stressors included in the job stressor index (job control, job demands, job insecurity, and effort-reward imbalance).

We then used fixed effects regression models to examine the extent to which the relationship between psychosocial job stressors and changes in the MHI-5 was due to within versus between person-related factors (such as gender or other individual factors such as personality, country of birth). Again, occupation was time invariant. This analysis was specifically conducted in relation to the two occupational groups of interest: carers and support workers, and health care workers. Fixed effects analyses are able to provide stronger evidence of the effect of psychosocial job stressors on changes in the mental health as they demonstrate the relationship between within-person changes in working conditions in relation to within-person changes in mental health. Hence, person-related factors including stable psychological factors are excluded from the models.

## 3 Results

The characteristics of those employed as carers is presented in Table 1. Overall, the reference group of other workers and carers and support workers had the worst working conditions. Table 1 also shows that carers and support workers had lower scores on the MHI-5 (mean = 73.79, 95% CI 73.41 to 74.16) than health care workers (mean = 76.82, 95% CI 76.40 to 77.20) or the reference group of other workers (mean = 75.76, 95% CI 75.64 to 75.88).

**Table 1:** Sample characteristics of those included in the random effects regression models, health and human care workers and other occupational groups, HILDA 2001 to 2016.

	Other workers	Carers and support workers	Health care workers	Human service workers
MHI (mean)	75.8	73.8	76.8	75.0
Income ($ mean)	50,564	42,620	60,397	48,425
Age (mean)	40.0	41.2	42.0	38.3

Psychosocial job stressors (%)				
No stressors	28.9	25.9	36.9	36.0
1 stressor	39.8	43.2	40.7	42.3
2 stressors	22.5	22.2	17.2	16.7
3 stressors	8.8	8.7	5.2	5.0

Gender (%)				
Male	72.6	14.6	21.4	33.0
Female	27.4	85.4	78.6	67.0

Employment arrangement (%)				
Permanent	55.8	57.7	66.5	58.1
Casual/Labour hire	16.4	26.2	11.5	24.1
Fixed-term	6.7	10.4	12.0	8.0
Self-employed	21.1	5.7	10.0	9.8

Education (%)				
Postgraduate	10.6	4.0	31.1	12.2
Bachelor	15.9	10.4	48.8	15.2
Diploma or certificate	36.0	55.6	10.2	26.5
High school	14.0	13.0	6.7	21.6
Below high school	23.5	17.0	3.2	24.6

Disability				
No	86.7	84.4	87.1	87.6
Yes	13.3	15.6	12.9	12.5

Country of birth				
Australia	79.5	79.6	75.6	82.5
Other English speaking	10.0	8.8	10.6	8.5
Non-English speaking	10.6	11.6	13.8	9.0

Household structure				
Couple without children	27.3	23.8	28.7	26.2
Couple with children	48.9	45.2	47.3	47.9
Lone parent with child	6.4	13.9	6.6	9.2
Lone persons	12.8	12.4	13.6	11.9
Multiple persons	4.6	4.7	3.8	4.8
Persons	14,108	2143	1165	12,111
Observations	66,020	7485	5682	51,757

**Fig. 2:**
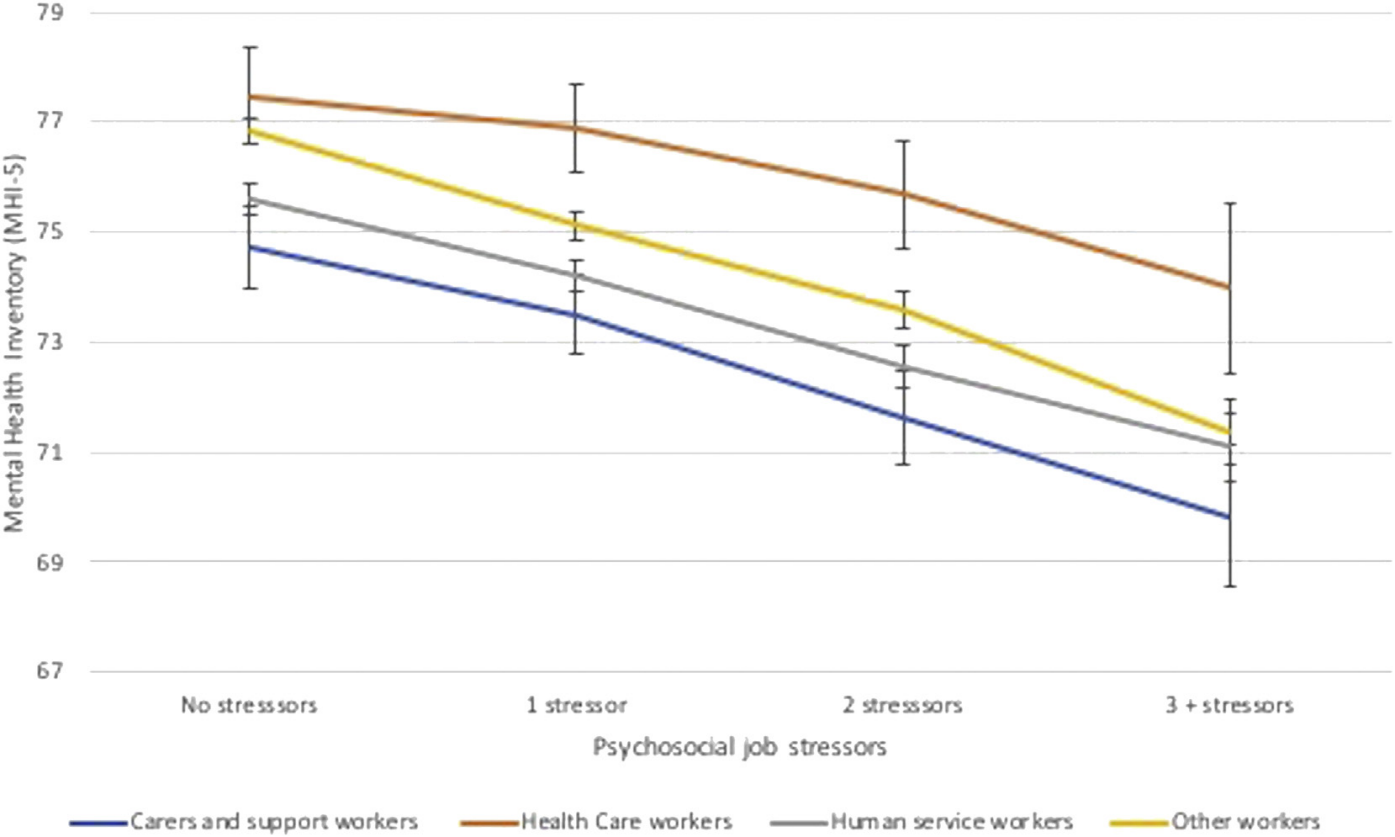
Marginal effects of psychosocial job quality on the mental health inventory (MHI-5), results for stratified random effects regression models, health and human care workers and other occupational groups, HILDA 2001 to 2016 Notes: Models adjust for age, gender, country of birth, household equivalized income, household structure, gender, employment arrangement, and education, and disability. Error bars represent 95% Confidence Intervals.

Results suggested that the relationship between psychosocial job stressors and mental health differed by occupational group (LR chi^2^ [9] = 47.07, *p* < 0.001) (regression results can be seen in Supplementary File 2). Based on this, models were stratified and the marginal effects of the psychosocial job stressors on mental health were calculated from random effects models (Fig. 2). In general, carers and support workers had an overall lower level of mental health (e.g., their baseline level of mental health was lower than the other workers). These workers had a 4.9-point decline in the MHI-5 from reporting no stressors to reporting three or more stressors (95% CI -6.23 to −3.57). Other human service workers also had low levels of mental health and experienced a 4.5 decline on the MHI-5 from reporting no stressors to 3 or more stressors (95% CI −5.13 to −3.87). Health care workers had the highest levels of mental health and experienced the smallest decline in the MHI-5 when moving from no stressors to three or more stressors (3.5 points, 95% CI −5.05 to −1.94).

We assessed the relationship between the individual components included in the psychosocial job quality index with mental health. This showed that job insecurity had the biggest effect on mental health across all occupational groups (Other workers: Coef. −3.73, 95% CI −4.01 to −3.44, *p* < 0.001; Carers and support workers: Coef. −4.16, 95% CI −5.07 to −3.26, *p* < 0.001; Health care workers: Coef. −3.08, 95% CI −4.53 to −1.64, *p* < 0.001; Human service workers: Coef. −3.59, 95% CI −3.96 to −3.22, *p* < 0.001). However, its effects were more apparent among carers and support workers. In most human service occupations, fairness of pay was associated with the next largest decline in mental health (Other workers: Coef. −2.11, 95% CI −2.38 to −1.85, *p* < 0.001; Carers and support workers: Coef. −1.98, 95% CI −2.69 to −1.27, p < 0.001; Human service workers: Coef. −1.63, 95% CI −1.92 to −1.34, p < 0.001) with the except of health care professions, where low control was associated with the second largest decline in mental health (Coef. −2.36, 95% CI −3.22 to -1.51, p < 0.001). Our analysis with the continuous measures of these scales showed results similar results to the binary measures.

We then conducted fixed effects regression models within our two (time-invariant) occupational groups of interest: carers and support workers and health care workers (Table 2). Results attenuated compared to random effects models. Compared to when a person employed as a carer or support worker reported no stressors, reporting three or more stressors resulted in a 3.07 (−4.56 to −1.59, *p* < 0.001) decline on the MHI-5. There was a 2.65 (95% CI −4.32 to −0.98, *p* = 0.002) decline on the MHI-5 when health care workers reported three or more stressors compared to when they did not report any stressors.

**Table 2:** The effect of psychosocial job stressors on the mental health inventory (MHI-5), results from fixed effects regression model, 2001 to 2016, HILDA.

	Carers and support workers			Health care workers		
	Coef.	95% CI	P value	Coef.	95% CI	P value
Psychosocial job stressors						
No stressors	0			0		
1 stressor	−0.64	−1.42–0.14	0.109	−0.30	−1.05–0.45	0.428
2 stressors	−1.95	−2.92–−0.99	<0.001	−1.19	−2.24–−0.15	0.025
3 stressors	−3.07	−4.56–−1.59	<0.001	−2.65	−4.32–−0.98	0.002
Constant	73.57	66.52–80.62	<0.001	74.57	68.07–81.07	<0.001
Persons	2143			1165		
Observations	7485			5682		

Notes: Coef. = Coefficient; 95% CI = Upper and lower confidence intervals at 95% significance; p value = statistical significance at 95%. Models control for age, income, employment arrangment, education, disability, household structure. Time invariant factors (gender, country of birth) drop out of the model.

## 4 Discussion

The results of this study suggest that lower skilled carers and support workers were more likely to be exposed to psychosocial job stressors than higher skilled health workers such as doctors and nurses. Results from the random effects regression also suggest that carers and support workers had poorer mental health than other workers and were more vulnerable to declines in their mental health when exposed to psychosocial job stressors.

Past research has argued that health-related caring or support employment is seen as feminized work (Huppatz, 2010), which is undervalued (Charlesworth, 2012) and underpaid (Hussein, 2017). It is also lower skilled work, particularly when compared to medical and other allied health professionals. From an occupational health perspective, there is considerable research to demonstrate the accumulation of psychosocial jobs stressors (as well as biological and chemical occupational exposures) in lower skilled, undervalued jobs (Landsbergis et al., 2014). In our analyses, those working as carers or support workers were mainly women who had lower income and education than either the reference group of other workers or compared to health care workers. Working women from lower socio-economic groups are traditionally conceptualized as having less agency, prestige or power in society (Landsbergis et al., 2014; Krieger et al., 2011; Krieger et al., 2008). Hence, it is likely that vulnerable groups select into caring and support work (Ahonen et al., 2018), where they are further exposed to work-related adversities such as insecure work (as evident in the fact that there was a greater proportion of casual workers in this group) and psychosocial job stressors.

Findings regarding the greater susceptibility to psychosocial job stressors in caring work can be explained by the “inverse hazards law” (Krieger et al., 2011; Krieger et al., 2008), which argues that the accumulation of health hazards tends to vary inversely with the power and resources of the populations affected. Even when they experienced no psychosocial stressors, mental health among carers and support workers was about 3-points lower than both the reference category of other workers and health care workers. Hence, people with lower levels of mental health may find themselves in working contexts where they experience even poorer health outcomes due to adverse occupational exposures. To test whether our results were in-fact driven by person-related selection effects, we conducted fixed effects regression models. Results of these models suggest that greater exposure to psychosocial job stressors was associated with declines in mental health, controlling for stable person-related factors (e.g., gender, country of birth, personality, etc.,) as well as those things that might vary over time, such as age, income, and employment arrangement. These findings give us confidence to argue that the poorer outcomes of carers and support workers is about more than health selection (e.g., different behavioural or lifestyle factors, as well as socio-other economic explanations for health status)–and is in fact related to their low-quality working environments.

While it is important to highlight the specific vulnerable group of carers and support workers, we also have to acknowledge that the entire category of health and human service workers were exposed to psychosocial job stressors and experienced declines in mental health in relation to this exposure, as seen in both random effects and fixed effects analysis. This is problematic for impacted individuals and their families because the experience of job stressors is undoubtedly associated with considerable distress, as well as sickness absence (Milner et al., 2015), turnover intentions (Van der Heijden et al., 2018; Gray and Muramatsu, 2013) and early retirement (Browne et al., 2018). However, in addition to these individual and organizational outcomes, the working conditions of health and human service workers may have flow-on effects to the quality of the care they provide to clients (Spence Laschinger and Leiter, 2006). In 2016, a review of 27 studies found that poor wellbeing among healthcare workers (e.g., depression, anxiety, poor quality of life and stress, and burnout) was significantly associated with poorer patient safety (Hall et al., 2016). This reinforces the likely importance of the relationship between psychosocial working conditions, the mental health of the health and human service workforce and outcomes for clients.

The limitations of this study are connected to the nature of the self-reported exposure and outcome data, which could lead to dependent misclassification. For example, unmeasured factors – such as attitudes or personality – could influence both a persons reporting of exposure and outcomes potential biasing the estimates in unknown ways. Another problem is that we do not have data on job stressors on emotional demands (Dollard et al., 2000; Madsen et al., 2010; van Vegchel et al., 2004), and workplace violence (Aagestad et al., 2016), which past research suggests are particularly prevalent among health and human service workers. We would encourage these measures to be incorporated into future studies. A further limitation concerns the fact that we were unable to disaggregate data any further than the two-digit level, thus obscuring effects that may be occurring within these categories. For example, the healthcare category combines data from both doctors and nurses, together with other health care workers such as allied health professionals. There is research to suggest that there are some important differences in psychosocial exposures in these professionals (Myhren et al., 2013). It is also notable that external generalizability of the study may be affected by drop out, although there was a relatively low proportion of missing data in the study. The weaknesses of the study may be offset by the strengths of the study, which include the strong methodological approach accounting for time invariant and time varying effects, longitudinal time frame of 16 waves, and cohort sample representing all occupational groups. Future areas of work could explore within-individual changes in these occupational categories thus exploring more dynamic changes in jobs over the life course. It is also important to consider the possible influence of interpersonal dynamics on the relationship between a person's reported job stressor exposures and mental health. It may be the case that workers in occupational groups embedded in hospital or other organisations are able to draw on collegial or supervisor support in times of stress. Thus, these workers may have better outcomes than those persons working as carers in home-based environment, where the employed people are more socially isolated from work colleagues. It is also important to consider client related factors influencing a worker's mental health, including length of time of the relationship. More investigation into the different dynamics of client and work-team related dynamics is potentially an interesting area of further work.

In conclusion, this study suggests there is differential exposure and susceptibility in the health and human care workforce. Carers and support workers were found to have greater exposure to psychosocial job stressors, and experienced notable declines in mental health in relation to these work exposures. There is a dire need for more investigation on working conditions among health and human service workers. Such research is critical to the design of future recommendations for workplace design, particularly as the number of people employed as health and human service worker is predicted to grow. For the workforce to be sustainable these jobs need to be structured so as to promote health and productivity and high quality of care.

## Author contributions

The article was conceived by AM, who also conducted analysis. TK and AM contributed to analytic design and to the interpretation of results. AM drafted the manuscript with feedback from all authors. All authors contributed to the final draft of the manuscript.

## Grants and/or financial support

AM was supported by a Victorian Health and Medical Research Fellowship. Further support provided by NHMRC Centre for Research Excellence: Centre of Research Excellence in Disability and Health (APP1116385) and NHMRC Partnership Project (APP1134499). The funding source had no involvement in the study design; collection, analysis and interpretation data; the writing of the report; or in the decision to submit the paper for publication.

## Conflict of interest

None.

## Appendix A Supplementary data

Supplementary tables 1 to 4 Image 1
