# Supplementary material for: The mental health impacts of health and human service work: Longitudinal evidence about differential exposure and susceptibility using 16 waves of cohort data
Source: Prev Med Rep. 2019 Feb 23;14:100826. doi: 10.1016/j.pmedr.2019.100826 (PMC6402427; doi:10.1016/j.pmedr.2019.100826)
Supplement: Supplementary file 1 — Supplementary tables 1 to 4 [file mmc1.docx]

Supplementary Table 1. Classification of healthcare workers

| Occupational group | ANZSCO code | Description of ANZSCO code |
| --- | --- | --- |
| Health aides and carers | 41  42 | Health and Welfare Support Workers  Carers and Aides |
| Health care workers | 25 | Health Professionals (allied health professionals, health diagnostic and promotion, health therapy, medical practitioners, nursing and midwifery) |
| Human service workers  *clerical and administration    *sales  *education  *hospitality  *legal  * protective services | 50-59  60-63  24  14, 43  27  44 | Clerical and Administration, Managers, Personal Assistants, General Clerical, Inquiry Clerks, Numerical Clerks, Other Clerical  Sales Workers, Sales Representatives, Sales Assistants, sales support  Education professionals  Hospitality managers, hospitality workers  Legal, Social and welfare professionals  Protective service officers |
| \| Other workers \|  \|  \| \| --- \| --- \| --- \| | All other codes |  |

Supplementary Table 2. Results of the random effects model, health and human service workers and other workers, interaction between psychosocial job stressors and occupational group (persons= 21,532, 130,944 observations)

|  | **Coef.** | **95% CI** | **P value** |
| --- | --- | --- | --- |
| **Psychosocial job stressors** |  |  |  |
| No stressors | 0 |  |  |
| At least one stressor | -1.64 | -1.77 - -1.51 | <0.001 |
| **Occupation** |  |  |  |
| Other workers | 0 |  |  |
| Carers and support workers | -0.60 | -1.20 - 0.01 | 0.055 |
| Health Care workers | 0.61 | -0.11 - 1.33 | 0.095 |
| Human service workers | -0.64 | -0.90 - -0.37 | <0.001 |
| **Psychosocial job stressors#Occupation** | |  |  |
| No stresssors#Other workers | 0 |  |  |
| At least one stressor#Carers and support workers | 0.20 | -0.19 - 0.58 | 0.322 |
| At least one stressor#Health care workers | 0.64 | 0.21 - 1.08 | 0.004 |
| At least one stressor#Human service workers | 0.21 | 0.02 - 0.41 | 0.033 |
| Constant | 76.08 | 75.85 - 76.31 | <0.001 |

Notes: Models adjust for age, gender, country of birth, household equivalized income, household structure, gender, employment arrangement, and education, and disability. Coef.= Coefficient; 95% CI = Upper and lower confidence intervals at 95% significance; p value= statistical significance at 95%.

Supplementary Table 3. The effect of psychosocial job stressors on the mental health inventory (MHI-5), results from random-effects regression model, 2001 to 2016, HILDA.

|  | Coef. | 95% CI | p value |
| --- | --- | --- | --- |
| Other workers |  |  |  |
| Psychosocial job stressors | | |  |
| No stressors | 0 |  |  |
| 1 stressor | -1.71 | -1.92 - -1.49 | <0.001 |
| 2 stressors | -3.25 | -3.56 - -2.93 | <0.001 |
| 3 stressors | -5.47 | -6.07 - -4.88 | <0.001 |
| Constant | 70.91 | 69.70 - 72.12 | <0.001 |
| Carers and support workers | | | |
| Psychosocial job stressors | | |  |
| No stressors | 0 |  |  |
| 1 stressor | -1.23 | -1.94 - -0.52 | 0.001 |
| 2 stressors | -3.11 | -3.99 - -2.24 | <0.001 |
| 3 stressors | -4.90 | -6.23 - -3.57 | <0.001 |
| Constant | 68.03 | 64.38 - 71.68 | <0.001 |
| Health Care workers |  |  |  |
| Psychosocial job stressors | | |  |
| No stressors | 0 |  |  |
| 1 stressor | -0.58 | -1.29 - 0.12 | 0.106 |
| 2 stressors | -1.79 | -2.77 - -0.82 | <0.001 |
| 3 stressors | -3.50 | -5.05 - -1.94 | <0.001 |
| Constant | 72.67 | 68.64 - 76.70 | <0.001 |
| Human service workers |  |  |  |
| Psychosocial job stressors | | |  |
| No stressors | |  |  |
| 1 stressor | -1.38 | -1.63 - -1.13 | <0.001 |
| 2 stressors | -3.05 | -3.41 - -2.69 | <0.001 |
| 3 stressors | -4.50 | -5.13 - -3.87 | <0.001 |
| Constant | 68.58 | 67.16 - 70.00 | <0.001 |

Notes: Models adjust for age, gender, country of birth, household equivalized income, household structure, gender, employment arrangement, and education, and disability. Coef.= Coefficient; 95% CI = Upper and lower confidence intervals at 95% significance; p value= statistical significance at 95%.
